# 

**Published:** 1901-04-15

**Authors:** 


### ANNOUNCEMENTS.

### ILLINOIS STATE DENTAL SOCIETY.

The thirty-seventh annual meeting will be held in Rockford, May 14th to 17th, inclusive. All members should make an effort to be present. The society is always glad to welcome reputable dentists, who are not members, from this and other States.

The local committee has arranged for an informal reception on Tuesday evening in the parlors of the Hotel Nelson. A short program has been prepared and all in attendance are cordially invited to be present and spend a sociable evening.

The following is the program as prepared by the executive committee: 1, The Annual Address by the President, Dr. J. G. Reid; 2, Report of Committee on Dental Science and Literature, Dr. A. W. Harlan; 3, Report of Committee on Art and Invention, Dr. Hart J. Goslee; 4, "Why Fillings Fail," Dr. T. F. Henry; 5, "Cement Anchorage for Fillings," Dr. J. J. Reed;

6, "Dental Jurisprudence," Edwin Hedrick, Esq., Peoria; 7, "Anomalous Cases of Malformed Teeth, with Suggestions as to Possible Causes," Israel P. Wilson, Burlington, Ia.; 8, "Preventive Dentistry," Garrett Newkirk, Los Angeles, Cal.; 9, "Air Chambers —Uses and Abuses," Dr. Alfred O. Hunt; 10, "Orthodontia"—Illustrated with Lantern Slides, J. N. McDowell; 11, "Submarine Gold" (Illustrated by Stereopticon Lantern), Dr. George A. McMillen; 12, "Antiseptic, Germicide and Disinfectant," Dr. A. H. Peck; 13, "The Physiological Function of Saliva," Dr. J. B. Dicus; 14, "Microbiology and Office Clinic," George D. Sitherwood; 15, "The Philosophy of Mastication—Relative to Artificial Dentures," Dr. B. J. Cigrand. In addition between forty and fifty clinics on practical up-to-date subjects are promised.

CLINICS.

1. A. W. Harlan, Chicago: Pyorrhœa Alveolaris.

2. J. W. Cormany, Mt. Carroll: Gold filling, using Bonwell's mechanical mallet.

3. J. E. Hancock, Joliet: Porcelain crown.

4. J. R. Callahan, Cincinnati, O.: Gold and tin in definite proportions for filling.

5. F. E. Roach, Chicago: Practical application of the wedge-lock facing.

6. C. C. Corbett, Edwardsville: Amalgam filling, cement anchorage.

7. C. DeWitt Lukens, St. Louis, Mo.: Orthodontia.

8. W. H. Taggart, Chicago: A. E. Peck's complete system for making porcelain inlay and restoration to absolutely fit the cavity.

9. F. H. McIntosh, Bloomington: A method of building up broken-down bicuspids and molars preparatory to receiving a gold crown.

10. W. T. Reeves, Chicago: Porcelain inlay or restoration. Exhibition of the Hammond electric furnace.

11. Truman W. Brophy, Chicago: Surgical clinic.

12. B. D. Wikoff, Chicago: A novel method of making a bridge.

13. E. H. Allen, Freeport: Gold filling.

14. A. W. McCandless, Chicago: (*a*) Setting crowns and bridges with gutta percha, using Kerr electric annealer to soften the gutta-percha; (*b*) alteration of plaster model, adapting ideal dental base plate to insure snugly fitting denture.

15. Thos. L. Gilmer, Chicago: Surgical clinic.

16. O. A. Chappell, Elgin: Application and use of dead soft gold.

17. W. V-B. Ames, Chicago: Subject to be announced.

18. Chas. P. Pruyn, Chicago: Pressure anesthesia for removal of a live pulp.

19. Josephine D. Pfeifer, Chicago: Filling root canals.

20. A. F. James, Oak Park: Ladmore and Bronson matrix.

21. Elliott R. Carpenter, Chicago: Pyorrhœa at root bifurcations and its treatment with oxyphosphate of copper.

22. D. M. Cattell, Chicago: Filling a proximo-occlusal cavity in a bicuspid or molar; gingival one-third with non-cohesive cylinders or mats; occlusal two-thirds, including step anchorage, with cohesive gold.

23. E. A. Royce, Chicago: Demonstration of the varying shades of the natural teeth in the same mouth as applied to artificial dentures.

24. Rob't Good, Chicago: Pyorrhoea Alveolaris; Younger's method.

25. C. N. Johnson, Chicago: Preparation of cavities.

26. S. F. Duncan, Joliet: Gold filling, using the Berry electric engine and mechanical mallet No. 2.

27. L. K. Stewart, Chicago: Long bites in continuous gum work.

28. W. W. Shryock, Fort Wayne, Ind.: Swedging seamless gold crown.

29. I. B. Crissman, Chicago: Labial cavity extending under the gum, filled with De Trey's gold, without rubber dam.

30. Geo. A. McMillen, Alton: Use of submarine gold.

31. A. O. Hunt, Chicago: Uses and abuses of air chambers, also W. E. Griswold's removable bridge.

32. A. G. Johnson, Chicago: A novelty for the assistance of annealing and carrying gold.

33. J. S. Bridges, Chicago: Porcelain inlay, demonstrating the Ash swedge.

34. C. B. Rohland, Alton: Subject to be announced.

35. J. H. Prothero, Chicago: Arrangement and occlusion of teeth

36. J. O. Brown, Chicago: Contour filling, using Watt's crystal gold.

37. J. W. Slonaker, Chicago: Nitrous oxide gas.

38. G. W. Dittmar, Chicago: Proximo-incisal cavity; step anchorage; showing cavity preparation and gold filling; using Rowan's cylinders and No. 20 extra cohesive foil.

39. W. F. Green, Evanston: Subject to be announced.

40. L. L. Davis, Chicago: Celluloid cement filling.

41. C. S. Case, Chicago: Restoration of facial contours by a combination of orthopedia and prosthesis; especially applicable for cleft palate patients.

42. C. B. Powell, Jacksonville: Treatment of root canals, demonstrating the use of a new broach holder of the clinitian's own design.

43. G. V. Black, Chicago: Demonstrations of mallet force, using tuptodynamomiter.

Signed,

C. R. TAYLOR, Streetor,
*Executive Committee.*

J. E. HINKINS, Chicago,
*Supervisor of Clinics.*

### OHIO, MICHIGAN AND INDIANA DENTAL MEETING.

The third triennial meeting of the State associations of Ohio, Michigan and Indiana, known as the original Tri-State Dental Meeting, will be held at the German House, corner of Michigan and New Jersey streets, Indianapolis, Indiana, June 4, 5 and 6, 1901, beginning at 10:00 A. M., Tuesday, June 4th. All practitioners who conduct their practices in a manner to command the respect of their fellow practitioners, are invited to attend and participate in the proceedings, whether they are members of a State association or not. These meetings are the largest and most interesting held in the United States. Fully eight hundred dentists will be present. The program includes some sixty clinics of great interest and importance. Railroad rates of a fare and a third for the round trip have been granted by the Central Traffic Association throughout the whole territory. For further information see the May journals or address, GEO. E. HUNT,
131 E. Ohio St., Indianapolis, Ind.

---

THE Board of Examiners of Pennsylvania will hold examinations simultaneously in Philadelphia and Pittsburg, May 7 to 10, 1901. Candidates should apply to Hon. James W. Latta, secretary Dental Council, at Harrisburg, Pa., for papers and information.

G. W. KLUMP, *Secretary*,
Williamsport, Pa.

---

THE New Jersey State Dental Society will hold its annual meeting at Asbury Park, July 17, 18, 19, 1901. The "Hornets" promise to be at home, and extend a hearty welcome to all visitors.

---

THE Oklahoma Board of Dental Examiners will meet at Oklahoma City, May 7th, at 10 A. M. for the purpose of examining applicants for license to practice.

E. E. KIRKPATRICK, *Secretary*.

---

THE Kentucky State Dental Association will hold its annual meeting in Louisville, May 14, 15, 16, 1901. The usual railroad and hotel rates have been secured.

---

THE Illinois, Kentucky and Indiana tri-state dental meeting will be held at Paducah, Ky., May 28, 29 and 30, 1901.

---

THE West Virginia Board of Dental Examiners will meet May 1, 2, 3, 1901, at Wheeling.

